# The potential impact of hematocrit correction on evaluation of tacrolimus target exposure in pediatric kidney transplant patients

**DOI:** 10.1007/s00467-018-4117-x

**Published:** 2018-10-30

**Authors:** Anne M. Schijvens, Fransje H. S. van Hesteren, Elisabeth A. M. Cornelissen, Charlotte M. H. H. T. Bootsma-Robroeks, Roger J. M. Brüggemann, David M. Burger, Saskia N. de Wildt, Michiel F. Schreuder, Rob ter Heine

**Affiliations:** 1 Radboud Institute for Molecular Life Sciences, Department of Pediatric Nephrology, Radboud University Medical Center, Amalia Children’s Hospital, P.O. Box 9101, 6500 HB Nijmegen, The Netherlands; 2 Radboud Institute for Health Sciences, Department of Pharmacy, Radboud University Medical Center, Nijmegen, The Netherlands; 3 Department of Pharmacology and Toxicology, Radboud University Medical Center, Nijmegen, The Netherlands; 4 Intensive Care and Department of Pediatric Surgery, Erasmus MC Sophia Children’s Hospital, Rotterdam, The Netherlands

**Keywords:** Therapeutic drug monitoring (TDM), Tacrolimus, Hematocrit, Pediatrics, Kidney transplantation

## Abstract

**Background:**

Tacrolimus is an important immunosuppressive agent with high intra- and inter-individual pharmacokinetic variability and a narrow therapeutic index. As tacrolimus extensively accumulates in erythrocytes, hematocrit is a key factor in the interpretation of tacrolimus whole blood concentrations. However, as hematocrit values in pediatric kidney transplant patients are highly variable after kidney transplantation, translating whole blood concentration targets without taking hematocrit into consideration is theoretically incorrect. The aim of this study is to evaluate the potential impact of hematocrit correction on tacrolimus target exposure in pediatric kidney transplant patients.

**Methods:**

Data were obtained from 36 pediatric kidney transplant patients. Two hundred fifty-five tacrolimus whole blood samples were available, together responsible for 36 area under the concentration-time curves (AUCs) and trough concentrations. First, hematocrit corrected concentrations were derived using a formula describing the relationship between whole blood concentrations, hematocrit, and plasma concentrations. Subsequently, target exposure was evaluated using the converted plasma target concentrations. Ultimately, differences in interpretation of target exposure were identified and evaluated.

**Results:**

In total, 92% of our patients had lower hematocrit (median 0.29) than the reference value of adult kidney transplant patients. A different evaluation of target exposure for either trough level, AUC, or both was defined in 42% of our patients, when applying hematocrit corrected concentrations.

**Conclusion:**

A critical role for hematocrit in therapeutic drug monitoring of tacrolimus in pediatric kidney transplant patients is suggested in this study. Therefore, we believe that hematocrit correction could be a step towards improvement of tacrolimus dose individualization.

**Electronic supplementary material:**

The online version of this article (10.1007/s00467-018-4117-x) contains supplementary material, which is available to authorized users.

## Introduction

Calcineurin inhibitors (CNIs) are the cornerstone of immunosuppressive therapy after kidney transplantation. Tacrolimus was introduced in the 1990s as an alternative to ciclosporin and is now widely used to prevent rejection after solid-organ transplantation in both adult and pediatric transplant recipients [1]. At the time of discharge after transplantation, approximately 70% of pediatric kidney transplant patients are treated with tacrolimus [2]. Tacrolimus is characterized by a narrow therapeutic index: high concentrations are associated with toxicity, malignancy, and infection, while low concentrations are associated with an increased risk of acute rejection [3, 4]. Furthermore, the intra- and inter-individual pharmacokinetic variability is high [1, 5]. Previously, body weight [6–8], *CYP3A5* polymorphisms [6–9], age [9, 10] and hematocrit level [6, 7] were found to have significant effects on pharmacokinetic variability in pediatric kidney transplant patients, especially in the early phase after transplantation. Due to the large pharmacokinetic variability, individualizing tacrolimus dosing regimens by performing therapeutic drug monitoring (TDM) to optimize the therapeutic effect and minimize adverse effects is essential and currently the standard of care.

For pediatric kidney transplant patients, little is known about the optimal targets for tacrolimus exposure. Furthermore, both whole blood trough concentrations and area under the concentration-time curves (AUCs) are used to adjust the tacrolimus dosing regimen of the individual patient. As it stands, it is unknown what the best pharmacokinetic parameter to predict treatment outcome is, and the relationship between blood trough concentrations and AUCs in pediatric patients remains a matter of debate [10]. As reference values for target exposure based on clinical trials in pediatric patients are lacking, adult targets are currently used [3, 11, 12]. Yet, large differences exist between the pediatric and adult population in terms of pharmacokinetics and physiology [13].

Hematocrit is a confounder for interpretation of tacrolimus exposure in whole blood. Several population pharmacokinetic studies have indeed identified hematocrit as a key factor for interpretation of tacrolimus whole blood concentrations in both the adult and pediatric population [6, 14, 15]. Low hematocrit results in lower whole blood exposure and can then be incorrectly interpreted as an increased apparent clearance of tacrolimus from whole blood, while the plasma concentrations and clearance remain unchanged [6, 7]. Currently, tacrolimus is generally measured as total concentrations in whole blood, whereas only the unbound concentration in plasma is pharmacologically active as it is available for cellular diffusion and distribution [16]. Measurement of plasma or unbound tacrolimus concentrations might therefore be a better reflection of the pharmacologically active drug, which is technically challenging and often unavailable in clinical practice [3, 17]. Tacrolimus extensively accumulates in erythrocytes, and the concentration in whole blood is the weighted average concentration of the plasma and erythrocyte fractions. Consequently, a change in hematocrit will affect the whole blood concentration, without affecting the pharmacologically active unbound plasma concentration [16, 18, 19].

Hematocrit values tend to change significantly in the first months after transplantation [14, 20]. Changes in hematocrit values can occur in both ways: most patients will have an increase in hematocrit as erythropoietin levels increase rapidly after a successful kidney transplantation. However, a decrease is also possible for both early after transplantation based on blood loss due to the surgery or dilution due to intensive fluid control and after discharge as a side effect of the frequently used concomitant immunosuppressive agent mycophenolic acid. As tacrolimus trough concentrations and AUCs are currently measured in whole blood, this may lead to incorrect dose adjustments and inadequate tacrolimus exposure [21].

The aim of our study was to evaluate the potential impact of hematocrit correction on tacrolimus target exposure in pediatric kidney transplant patients.

## Methods

### Study design

To evaluate the impact of hematocrit correction on tacrolimus dose individualization in pediatric kidney transplant patients, we performed a retrospective cohort study at our tertiary referral center (Radboudumc Amalia Children’s Hospital, Nijmegen) in the Netherlands.

### Setting and subjects

Pediatric kidney transplant patients (aged 1–18 years) undergoing therapeutic drug monitoring for tacrolimus between 2012 and 2017 were included when an AUC_0–12 h_ or AUC_0–8 h_ was available. A subset of our study population has previously been described by Martial et al. [22]. Patients were excluded in cases where a hematocrit value was not available within 2 days of sampling. Sampling for the AUCs was typically performed at the following time points: pre-dose and 1, 2, 3, 4, 8, and 12 h after tacrolimus intake. All patients received oral capsules (Prograft, Astellas Pharma) or a suspension (as extemporaneous preparation). Tacrolimus doses were adjusted based on TDM to achieve the whole blood target exposure that depends on time post transplantation and the immunosuppressive regimen of choice, as shown in Table 1 and Table 2 [3, 11, 12, 23, 24]. In our hospital, two treatment regimens with tacrolimus are commonly used: a prednisolone-free immunosuppressive regimen (according to the TWIST protocol) [25] or a triple therapy including prednisolone and mycophenolate mofetil [3]. As food may decrease the rate and extent of tacrolimus absorption, patients were instructed to always take the medication in an identical manner (either with or without food). The Ethics Committee of the Radboud University Medical Center waived the need for ethical approval according to the Dutch Law on Human Research, as only patient chart data were collected.

**Table 1:** Target whole blood concentrations and predicted plasma concentrations for tacrolimus using different hematocrit values

	Time post-transplantation	Target range wb^a^ (μg/l)	Predicted target range plasma (μg/l)	Predicted target range wb (μg/l)	Predicted target range wb (μg/l)
		literature Ht = 0.35	literature Ht = 0.35	Ht = 0.30	Ht = 0.25
Target trough level (*C*_0_) prednisolone-free regimen	0–4 weeks	10–20	0.27–0.58	8.6–17.2	7.2–14.5
	4 weeks–6 months	5–15	0.13–0.42	4.3–12.9	3.6–10.8
	6–12 months	5–10	0.13–0.27	4.3–8.6	3.6–7.2
	> 12 months	4–8	0.10–0.21	3.4–6.9	2.9–5.8
Target trough level (*C*_0_) immunosuppressive regimen including prednisolone	0–4 weeks	10–15	0.27–0.42	8.6–12.9	7.2–10.8
	4 weeks–6 months	7–12	0.19–0.33	6.0–10.3	5.1–8.7
	6–12 months	5–10	0.13–0.27	4.3–8.6	3.6–7.2
	> 12 months	4–8	0.10–0.21	3.4–6.9	2.9–5.8

*AUC* area under the concentration time curve, *C*_0_ trough concentration, *wb* whole blood

^a^Targets according to our local hospital protocol which is based on the following references: [23, 3, 24, 11]

**Table 2:** Target whole blood AUCs and predicted plasma AUCs

	Time post-transplantation	Target wb AUC_0–12 h_ (range)^a^	Target plasma AUC_0–12 h_ (range)
Target AUC_0–12 h_	0–6 weeks	210 h μg/l (168–252)	6.9 h μg/l (5.4–8.4)
	> 6 weeks	125 h μg/l (100–150)	4.0 h μg/l (3.1–4.8)

*AUC* area under the concentration time curve, *wb* whole blood

^a^Targets according to our local hospital protocol which is based on the following references: [3, 8]

Step 1: Derivation of hematocrit corrected target exposure

As tacrolimus dose individualization in children is guided by measurement of whole blood concentrations [3], we derived the associated plasma trough concentrations and plasma AUCs to use as hematocrit corrected target exposure. Whole blood concentrations (*C*_wb_) can be calculated from plasma concentrations, using the fraction of hematocrit (*f*_HCT_) for weighting with the following equation (Eq. 1), in which *B*_max_ is the maximum binding concentration of 418 μg/L and *K*_d_ is the dissociation constant of 3.8 μg/L [15]:

$$ {C}_{\mathrm{wb}}={C}_{\mathrm{p}}\bullet \left(1+\frac{B_{\mathrm{max}}\bullet {f}_{\mathrm{HCT}}}{C_{\mathrm{p}}+{K}_{\mathrm{D}}}\right) $$

Equation 1 can be rearranged to calculate the plasma concentrations if the whole blood concentration and fraction hematocrit are known, with the following equation (Eq. 2):

$$ {C}_{\mathrm{p}}=\frac{C_{\mathrm{wb}}-{K}_{\mathrm{D}}-{B}_{\mathrm{max}}\bullet {f}_{\mathrm{HCT}}+\sqrt{{\left({B}_{\mathrm{max}}\bullet {f}_{\mathrm{HCT}}+{K}_{\mathrm{D}}-{C}_{\mathrm{wb}}\right)}^2+4\bullet {C}_{\mathrm{wb}}\bullet {K}_{\mathrm{D}}}}{2} $$

For calculation of the plasma target concentrations we assumed a *f*_HCT_ of 0.35 L/L in the adult population, based on hematocrit values found in previous studies conducted in adult kidney transplant recipients [14, 26–28].

#### Converted plasma target trough concentrations

**Fig. 1:**
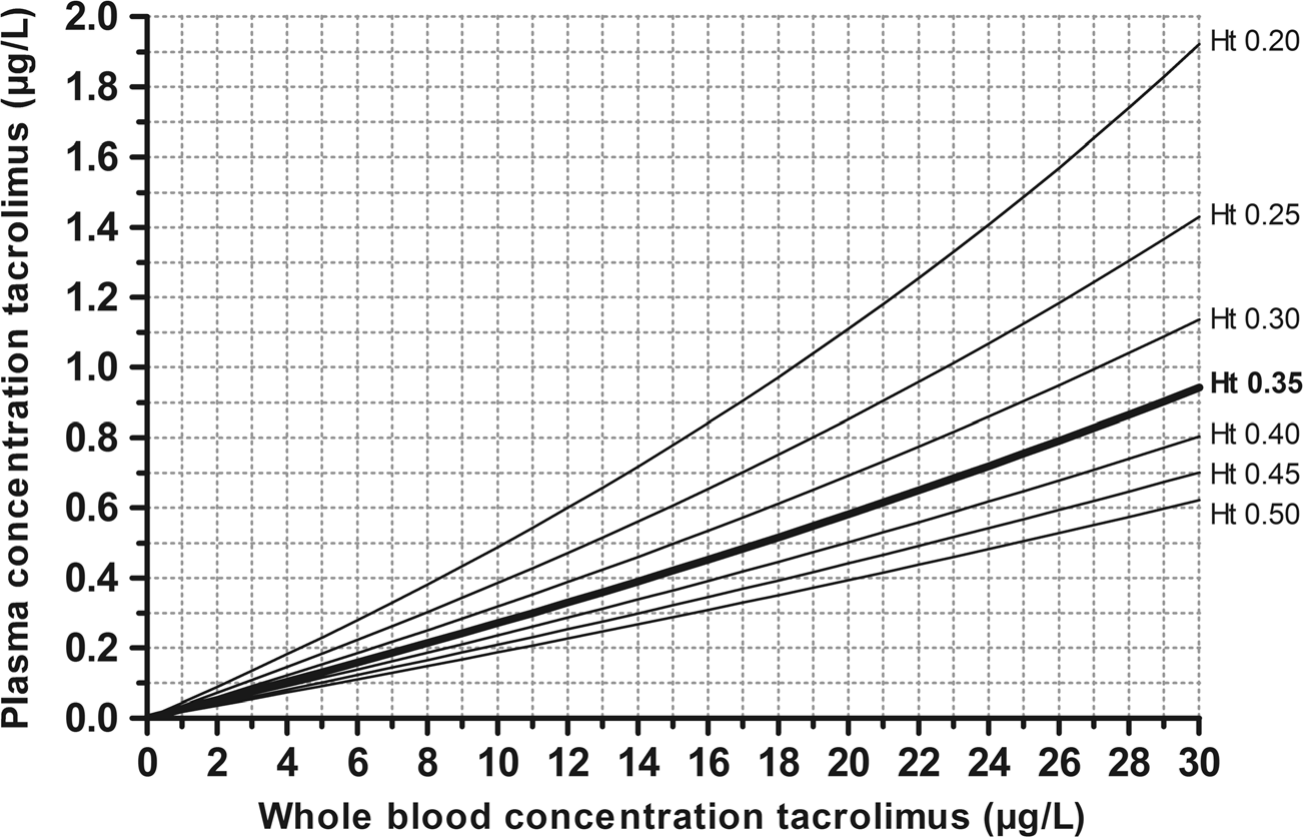
Nomograph of predicted tacrolimus plasma trough concentrations corrected for hematocrit

Subsequently, whole blood target trough concentrations were converted to plasma target trough concentrations using the aforementioned Eq. 2. The target ranges for converted plasma trough concentrations and whole blood trough concentrations with different hematocrit values are shown in Table 1. To further illustrate the influence of hematocrit, a nomograph (Fig. 1) is created using Eq. 2, in which the relationship between whole blood tacrolimus concentrations and plasma tacrolimus concentrations for different hematocrit levels is shown.

#### Converted plasma target AUCs

Whole blood target AUCs cannot be directly converted to plasma target AUCs, due to the non-linear binding of tacrolimus and thus a varying blood-to-plasma ratio during a dosing interval. Therefore, the individual whole blood concentrations on which the target AUCs are based should be converted to plasma concentrations, and subsequently, the plasma target AUC can be calculated. In the literature, however, these individual whole blood concentrations for the AUC targets are not available. Therefore, we simulated the steady-state whole blood pharmacokinetics of tacrolimus in 1000 virtual adult patients, based on a previously validated population pharmacokinetic model for tacrolimus [29] with the software package NONMEM V7.4.1. For calculation of the AUCs, we integrated the predicted whole blood concentrations and plasma concentrations versus time during a 12-h dosing interval for all 1000 individuals. A power trend line fitted our data best (*R* squared coefficient of 0.93, Supplementary Fig. 1). Subsequently, plasma target AUCs were calculated using the corresponding formula *y* = 0.0218*x*^1.0772^, where *y* and *x* represent the plasma target AUC and whole blood target AUC, respectively. The target ranges for whole blood AUCs and converted plasma AUCs are shown in Table 2.

Step 2: Derivation of hematocrit corrected trough concentrations and AUCs of the patients

Whole blood trough concentrations of the patients were converted to plasma trough concentrations using the aforementioned Eq. 2. Whole blood AUCs were converted to plasma AUCs by converting the individual concentrations of different time points on which the total AUC_0–12 h_ was based.

Step 3: Evaluation of target exposure

Subsequently, whole blood AUCs and trough concentrations of the patients as well as predicted plasma AUCs and trough concentrations of the patients were compared with the corresponding target concentrations.

Step 4: Evaluation of differences in interpretation of target exposure

Finally, to evaluate the impact of hematocrit correction in TDM of tacrolimus, differences in the interpretation of target exposure were identified by comparing the evaluation of target exposure for whole blood concentrations with and without hematocrit correction.

### Data collection

A validated liquid chromatography-tandem mass spectrometry (LC-MS/MS) bioanalytical assay was used to determine tacrolimus whole blood concentrations. The range of the assay was 1–300 μg/L. Intra-assay precision and accuracy was 3.4%, 2.2%, 3.0% and 102%, 94%, and 94%, respectively, at 3.04, 6.23, and 13.0 μg/L (*n* = 6), respectively [22, 30]. Furthermore, the following baseline characteristics were collected from the electronic patient records: age at kidney transplantation, gender, ethnicity, time post transplantation, donor type (living or deceased), height, and body weight. In addition, several laboratory data were collected: hematocrit, blood hemoglobin, serum creatinine, and serum urea. The estimated glomerular filtration rate (GFR) was calculated with the adapted Schwartz formula (*K* × height (cm)/serum creatinine (μmol/L)) with a *k* value of 36.5 [31]. Variables missing on the day of sampling were obtained by selecting the value closest to this date with a maximum for hematocrit of 2 days.

### Data analysis

Baseline variables were summarized using median and interquartile range (IQR). Whole blood trough concentrations were evaluated using the target concentrations according to our local hospital protocol. As shown in Table 1, target concentrations vary with time after transplantation and concomitant immunosuppressive medication [3, 11, 23, 24]. Furthermore, the whole blood AUCs were compared to the AUC_0–12 h_ target range of 210 ± 20% μg h/L up to 6 weeks post-transplantation and 125 ± 20% μg h/L upon 6 weeks post-transplantation (Table 2) [3, 12]. The AUCs_0–12 h_ were calculated using the linear-log trapezoidal method. To adequately compare the individual AUCs to the target AUC, the estimated AUC_0–8 h_ was extrapolated to an AUC_0–12 h_.

## Results

### Study population

A total of 37 children (age range 1.8–17.1 years) were eligible for inclusion in this study. One patient was excluded because the closest hematocrit value was 18 days before sampling. Data on a total of 255 tacrolimus whole blood concentrations were available, together providing 36 AUCs. Patient characteristics are presented in Table 3. The AUCs and trough levels were measured at any time post transplantation, predominantly in the first 2 weeks after transplantation. Of note, 33 of the 36 patients (92%) had a lower hematocrit value (median 0.29) than the reference hematocrit value of adult kidney transplant patients of 0.35 L/L on which the current recommendations of the dosing guidelines are based.

**Table 3:** Patient characteristics

	*n* (%)	Median	IQR
Number of study participants	36		
Age at time of transplantation (years)		8.3	4.3–14.9
Gender (*n*)			
Male	21 (58)		
Female	15 (42)		
Ethnicity (*n*)			
Caucasian	34 (94)		
African	2 (6)		
Time post transplantation (days)		12	9–13
Donor			
Living	26 (72)		
Deceased	10 (28)		
Height (cm)		124.4	95.0–160.9
Total body weight (kg)		23.1	15.7–46.2
Laboratory measurements			
Hematocrit (l/l)		0.29	0.26–0.31
Blood hemoglobin (mmol/l)		6.2	5.5–6.7
Serum creatinine (μmol/l)		55	37–103
Serum urea (mmol/l)		6.4	5.1–10.4
eGFR (ml/min/1.73 m^2^)		85	52–123

*eGFR* estimated glomerular filtration rate

### Step 1 and 2

**Fig. 2:**
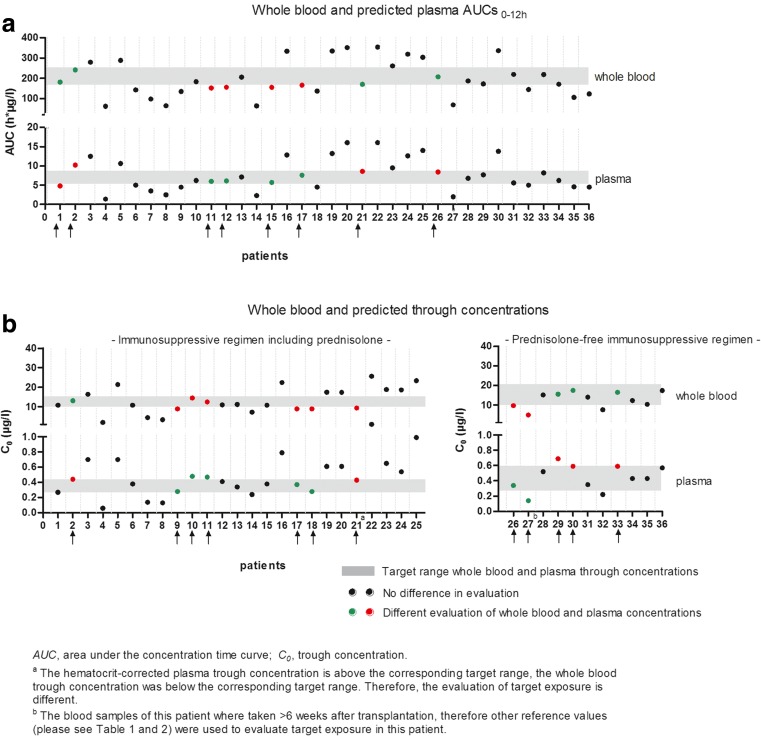
Whole blood and predicted plasma AUCs_0–12 h_ and trough concentrations compared to the target whole blood and predicted target plasma concentrations

For every individual patient, a whole blood AUC_0–12 h_ (range 62–354 h μg/L) and whole blood trough concentration (range 2.0–25.7 μg/L) were converted to the corresponding plasma AUC_0-12h_ (range 2.0–16.0 h μg/L) and plasma trough concentration (range 0.06–1.02 μg/L). Figure 2 shows the target AUCs and target trough concentrations, whole blood and predicted plasma AUC_0–12 h_, and whole blood and predicted plasma trough concentration of the individual patient.

### Step 3

Furthermore, Fig. 2 shows the evaluation of the AUCs and trough concentrations of the patients when compared to the corresponding target whole blood and predicted plasma ranges. Of the whole blood AUCs, 69% (25/36) were outside the target range, compared to 58% (21/36) of the whole blood trough concentrations. For the predicted plasma AUCs and trough concentrations, similar numbers were found (69% and 61%, respectively).

### Step 4

Using the hematocrit corrected target levels, a different evaluation for target exposure for either the trough level, AUC or both, was defined in a total of 42% (15/36) of our pediatric kidney transplant patients. In Table 4, the number of whole blood and predicted plasma values in and out of range are shown. For the AUCs, a different evaluation of target exposure was found in 22% (8/36) of the cases. For the trough concentrations, a different evaluation was shown in 33% (12/36) of the patients.

**Table 4:** Number of whole blood and predicted plasma AUCs and trough concentrations in and out of range

	AUC_0–12 h_ wb in range	AUC_0–12 h_ wb out of range	*C*_0_ wb in range	*C*_0_ wb out of range
Plasma—in range	7	4	9	5
Plasma—out of range	4	21	6	16
Total	11	25	15	21

*AUC* area under the concentration time curve, *C*_0_ trough concentration, *wb* whole blood

## Discussion

Tacrolimus is a key immunosuppressive agent in the majority of pediatric kidney transplant patients. This study shows the importance of hematocrit correction in tacrolimus target evaluation in this population. Using hematocrit corrected target concentrations, a different interpretation of tacrolimus exposure was found in 42% of our patients. As tacrolimus has a narrow therapeutic index, indicating that small variations in drug exposure can have a relevant impact on graft survival and toxicity, the results of this study suggest that using hematocrit corrected targets could prevent incorrect dose adjustments of tacrolimus based on whole blood concentrations. As the majority of our patients had a lower hematocrit value than the reference value of adult kidney transplant patients of 0.35 L/L, which causes an underestimation of the pharmacologically active concentration, toxicity in particular may be prevented.

Currently, therapeutic target ranges for tacrolimus exposure in pediatric kidney transplant patients are based on empirical observations in adult transplant patients, as reference values based on clinical trials in pediatric patients are lacking [3, 12]. Especially at the time of kidney transplantation, hematocrit values are generally low and tend to change significantly in the first months after transplantation. This underlines the need to take this into account in the TDM of kidney transplant patients [14, 20]. Moreover, this study shows that in our population hematocrit levels are significantly decreased and effort should be made to adequately correct hematocrit in these patients.

The importance of hematocrit correction in adult kidney transplant patients has been highlighted previously [14, 27, 32]. Størset et al. developed a population pharmacokinetic model for tacrolimus dosing in kidney transplant patients and recommend to standardize tacrolimus whole blood concentrations to a hematocrit of 45% to reflect the unbound (active) drug more closely, showing that hematocrit is a confounder and not a covariate for tacrolimus pharmacokinetics [14, 27]. Furthermore, a pharmacokinetic study conducted by De Jonge et al. indicated that hematocrit explained 4–14% of variability in tacrolimus pharmacokinetic parameters [32].

The challenge for clinicians to attain the optimal target exposure in the individual patient after kidney transplantation has been previously described by Ekberg et al. and Størset et al., who found 50% and 42%, respectively, of tacrolimus trough concentrations outside the target range during the first 6–8 weeks post transplantation [14, 33]. Our data show even higher percentages of AUCs and trough concentrations outside the proposed target range, underlining the large inter-individual variability and challenge for clinicians to attain the optimal target concentration.

To our knowledge, this is the first study investigating the role of hematocrit in the interpretation of tacrolimus whole blood exposure in pediatric kidney transplant patients in clinical practice. As the optimal strategy for TDM is still under debate, both AUC and trough concentrations are currently used in the clinical setting. Reported correlations between tacrolimus AUC and trough concentration are variable, indicating that trough concentrations alone may be a poor predictor of exposure [34]. A pharmacokinetic study previously conducted in a small subset of our study population, recommends the use of AUC as a driver for dose adaptations rather than trough concentrations in very young pediatric kidney transplant patients [22]. One of the strengths of this study is that both AUC and trough concentrations were evaluated to make the results as broadly applicable as possible. Unfortunately, there is no consensus among transplant centers on the optimal tacrolimus target exposure [1]. The target concentrations in our center are based on European consensus guidelines [3]; however, as target concentrations vary among centers, the predicted plasma target concentrations should be adjusted according to local clinical practice.

An important limitation of our study is its retrospective design, which may cause information bias. Although the observed whole blood concentrations were evaluated retrospectively, the exact time of dose administration was known in 35/36 of our patients. For one patient, the approximate time of dose administration was recorded in the medical file. As all patients were admitted to the hospital during the time of sampling and medication was administered by the nursing staff, we believe that the adherence to the medication is good and therefore full compliance was presumed. Due to the retrospective design of this study, laboratory values of 15 patients were unavailable on the day of sampling; therefore, these variables were imputed by choosing value closest to the day of sampling with a maximum of 2 days for hematocrit. This, however, is a limitation of our study and ideally all samples would be paired.

In this study, values for the binding capacity (*B*_max_) and affinity constant (*K*_d_) were obtained from a previously conducted study in adult liver transplant patients [15]. In addition, Zahir et al. found similar values for *B*_max_ and *K*_d_ in 40 liver transplant recipients using the same equation [35]. Recently, Størset et al. also used Eq. 2 to estimate the pharmacokinetic disposition parameters to develop a theory-based population pharmacokinetic model of tacrolimus in adult kidney transplant patients [27]. As our study involves pediatric kidney transplant patients, these values should ideally be obtained from this specific population by determination of the blood:plasma ratios of tacrolimus concentrations in pediatric kidney transplant recipients using the equation previously described by Piekoszewski et al. and Jusko et al. [15, 36].

As previously mentioned, the confounding effect of hematocrit variability can have a significant impact on the evaluation of tacrolimus whole blood concentrations as tacrolimus is highly bound to erythrocytes. As 99% of tacrolimus in plasma is bound to proteins, mainly albumin and α-1-acid glycoprotein, this could hold true for variations in albumin concentrations as well [1]. Although previous research showed no influence of albumin on whole blood tacrolimus concentrations in adult kidney transplant patients [14], this should be evaluated in pediatric kidney transplant patients as well. The influence of albumin was not evaluated in the current study, because on the day of sampling albumin concentrations were available for only eight of our patients. Furthermore, due to low patient numbers and tight monitoring, “hard” clinical endpoints (e.g., toxicity, rejection, or infection) could not be identified in our study population.

For pediatric kidney transplant recipients, long-term graft survival is especially important. Clinical trials in pediatric kidney transplant patients to obtain the optimal target exposure are however scarce [3]. As it is difficult to conduct clinical trials with “hard” clinical endpoints in small patient groups, we feel that all available information should be used to optimize TDM in pediatric patients. Although technically challenging, measuring both total and unbound tacrolimus concentrations in the plasma of pediatric kidney transplant patients would be an important opportunity for future research. Nonetheless, as tacrolimus is known to show high affinity for erythrocytes, we advocate a critical role for hematocrit correction in TDM of tacrolimus in pediatric kidney transplant patients. We consider the current study a proof-of-concept that hematocrit correction may be of added value in dose individualization of tacrolimus, especially in the pediatric population. In future studies, therefore, effort should be made to characterize *B*_max_ and *K*_d_ in pediatric kidney transplant patients and to prospectively investigate the impact of hematocrit correction on clinical endpoints.

## Electronic supplementary material

Supplementary Figure 1 Simulation whole blood AUCs and corresponding plasma AUCs (PNG 227 kb)

High Resolution Image (TIF 128 kb)
