# Supplementary figures and images for: The potential impact of hematocrit correction on evaluation of tacrolimus target exposure in pediatric kidney transplant patients
Source: Pediatr Nephrol. 2018 Oct 30;34(3):507–15. doi: 10.1007/s00467-018-4117-x (PMC6349786; doi:10.1007/s00467-018-4117-x)

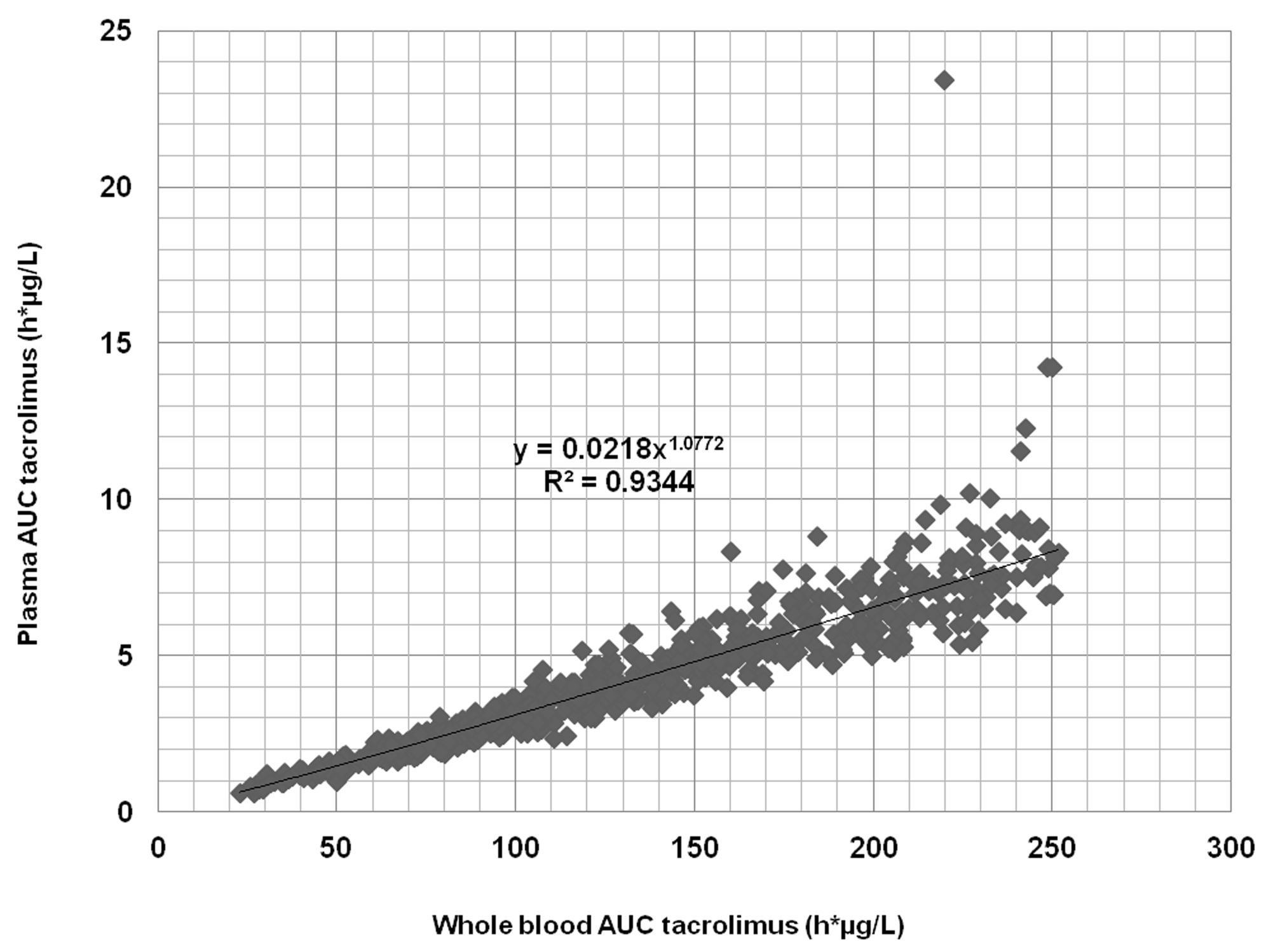

Supplement: Supplementary file 1 — Simulation whole blood AUCs and corresponding plasma AUCs (PNG 227 kb) [file 467_2018_4117_Fig3_ESM.png]

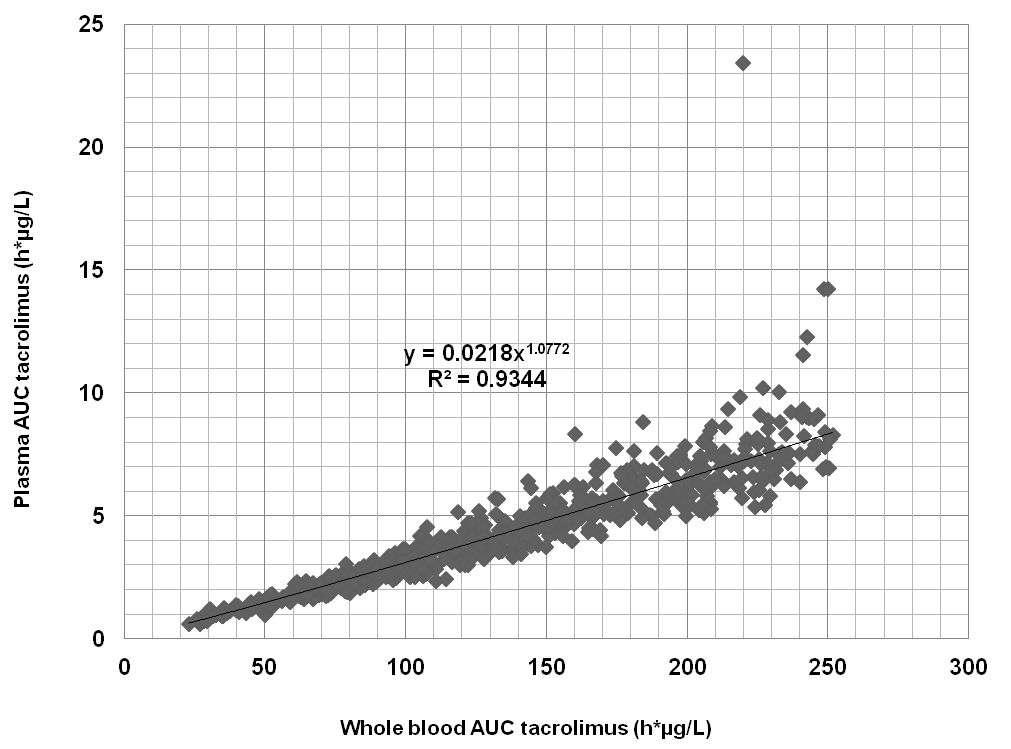

Supplement: Supplementary file 2 — High Resolution Image (TIF 128 kb) [file 467_2018_4117_MOESM1_ESM.tif]
